# Management and Outcomes of Kidney Transplant Candidates With Severe Pulmonary Hypertension: A Single-center Strategy and Experience

**DOI:** 10.1097/TXD.0000000000001640

**Published:** 2024-05-16

**Authors:** Dhiren Kumar, Nihar Raju, Bhupinder Prajapati, Irfan Moinuddin, Shreyank Tripathi, Daniel Grinnan, Deepak Thomas, Gaurav Gupta

**Affiliations:** 1 Virginia Commonwealth University, Internal Medicine, Richmond, VA.; 2 Richmond Nephrology Associates, Nephrology, Richmond, VA.

## Abstract

**Background.:**

Severe pulmonary hypertension (PH) is associated with high mortality posttransplant and thus is considered a contraindication to kidney transplantation. In this study, we describe the pretransplant management and posttransplant outcomes in patients with severe PH using a multidisciplinary approach.

**Methods.:**

Between 11 of 2013 and 8 of 2022, we identified all patients with severe PH on initial pretransplant workup who underwent ultrafiltration (UF) or medical therapy for PH before transplant. Posttransplant we evaluated the perioperative course, renal function, graft, and patient survival. We compared survival to those who remained waitlisted or were delisted.

**Results.:**

Three-two patients (mean age = 55.03 ± 10.22 y) diagnosed with severe PH on pretransplant screening echocardiogram. Thirty patients (94%) were subjected to a median of 4 (range, 3–8) UF sessions with an average weight loss of 4.33 ± 2.6 kg. Repeat assessment of PH revealed a decline in mean pulmonary artery systolic pressure from 67 ± 12 mm Hg to 43 ± 13 mm Hg (*P* < 0.0001). Seventeen patients (53%) received a kidney transplant. The mean estimated Glomerular Filtration Rate at 3, 6, 9, and 12 mo was 72 ± 27, 72 ± 28, 75 ± 29, and 75 ± 29 mL/min/1.73 m^2^. Among, those who underwent transplantation both graft and patient survival was 100% at 1-y posttransplant. Overall, since the UF intervention, at a median follow-up of 88 ± 12 mo those transplanted had a patient survival of 88% while those who remained on dialysis had a survival of 53% (*P* = 0.0003).

**Conclusion.:**

In this single-center study, we report postcapillary PH can be a significant contributor to elevations in pulmonary artery systolic pressure. Using a multidisciplinary approach, PH can improve with volume removal and phosphodiesterase 5 inhibitors therapy leading to a successful posttransplant outcome.

The presence of pulmonary hypertension (PH) in the end-stage renal disease (ESRD) population is a triple threat. First, it is a common occurrence which increases in incidence over time; secondly, it carries an extremely poor prognosis in this population and finally it remains an orphan disease without well established guidelines for effective therapy in either the nephrology, kidney transplant, or pulmonary literature.

The incidence of PH in the dialysis population is high with almost a third of the patients exhibiting some degree of PH while on dialysis.^1^ Further, there seems to be a graded positive relationship with dialysis vintage and PH prevalence and severity with up to 50% prevalence in patients with a dialysis vintage of greater than 2 y.^2^ This is particularly relevant as increasing numbers of dialysis patients with prolonged vintage are being referred for kidney transplant in the context of recent U.S. policy changes.^3^

The mortality associated with PH in the chronic kidney disease (CKD) population also remains exceedingly high. In a recent meta-analysis by Tang et al,^4^ the presence of PH was an independent risk factor for all-cause mortality with an increase in relative risk as CKD progresses peaking with patients on dialysis (RR, 2.08; 95% CI, 1.35-3.20; *P* = 0.001). The authors of this meta-analysis postulated that volume overload is a confounder in this population and cannot be accounted for in prognostic studies. It is plausible, that patients with or without volume overload may be clubbed together into the severe PH category and declined for a life-saving kidney transplant.

Although the ACC/AHA scientific statement on cardiac disease evaluation in kidney transplant recipients recognizes the need to consider patients with PH for transplant, no specific recommendations are provided beyond referral to a PH expert.^5^ In the PH literature these patients are commonly classified into group 5 PH. This group consists of a very heterogeneous population that has traditionally been excluded in PH therapeutic trials. In the ESRD population, these causes could be a combination of chronic volume overload, high output related to arteriovenous fistula-associated shunting, or the poorly defined renal failure associated vascular calcification and endothelial dysfunction.^6^

In most cases, the latter 2 causes do not lend themselves to effective intervention. The arteriovenous fistula remains a lifeline for these patients and ligation is only an option after successful transplantation. Therefore, we hypothesized “a priori” that chronic volume overload may be the most important modifiable variable in kidney transplant candidates with severe PH, followed by subsequent referral for targeted pulmonary vasodilator therapy to optimize a patient’s kidney transplant candidacy.

Herein, we report our pretransplant approach to patients with severe PH, the posttransplant outcomes of those patients who received a kidney transplant including graft function, delayed graft function rate, postoperative intensive care unit (ICU) stay, 30-d readmissions, graft function, graft, and patient survival while comparing patient survival to those who remain on dialysis.

## MATERIALS AND METHODS

In a prospective single-center protocol, all kidney transplant candidates referred to our center between November 2013 and August 2022 were assessed for inclusion if they had a diagnosis of nonvalvular severe PH on echocardiogram. A multidisciplinary team that included a transplant nephrologist (D.K.), cardiologist (D.T.), and PH expert (D.G.) evaluated all patients.

**Figure 1.**
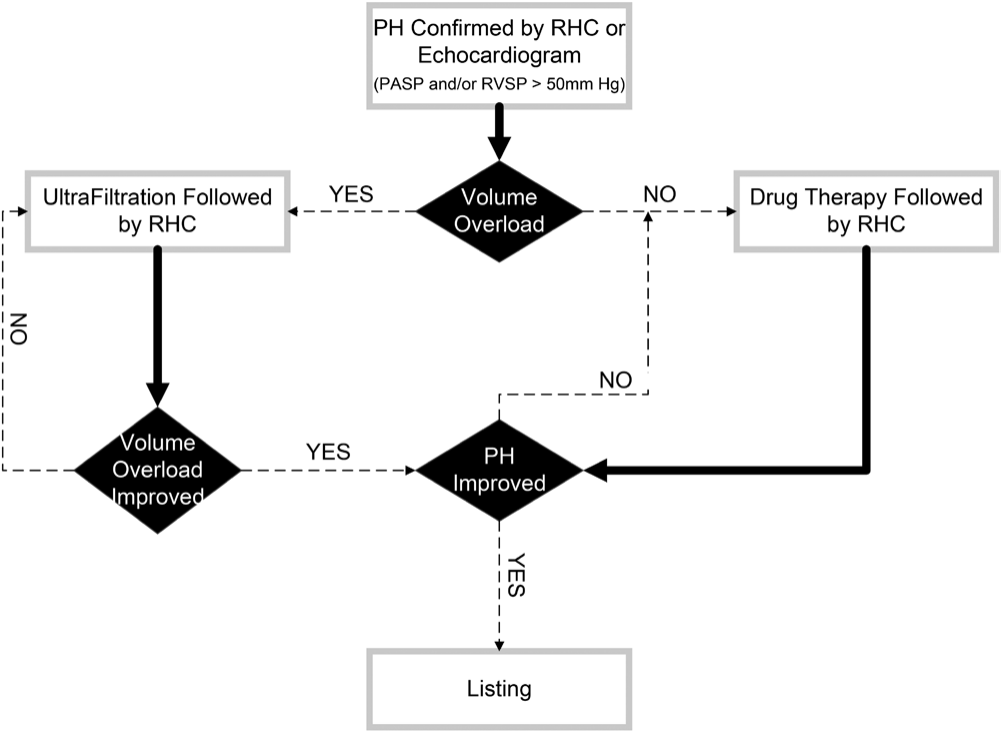
Flowchart of management. PASP, pulmonary artery systolic pressure; PH, pulmonary hypertension; RHC, right heart catheterization; RVSP, right ventricular systolic pressure.

Those with volume overload (RA pressure > 6 mm Hg or PCWP > 12 mm Hg) and moderate to severe PH (pulmonary artery systolic pressure [PASP] or right ventricular systolic pressure [RVSP] > 50 mm Hg) evidenced on echocardiogram itself or required confirmation by right heart catheterization (RHC) underwent aggressive UF. Patients underwent either daily UF as an inpatient or aggressive UF as an outpatient, followed by RHC, after presumptive achievement of a new dry weight based upon the assessment of the primary author (D.K.). If this initial step did not resolve severe PH or it was initially determined that PH was not related to volume (PASP or RVSP > 50 mm Hg with RA pressure < 6 mm Hg or PCWP < 12 mm Hg) then the patients were referred to a PH specialist for targeted therapy. Figure 1 outlines our approach. Pre-UF and Post-UF RHC parameters were compared using a paired t-test (MedCalc, Version 22.006). Number of sessions of UF required, change in weight, and number of blood pressure medications were compared.

The patients were then followed on dialysis, on the waitlist, or after kidney transplant, and their outcomes were monitored. Of those who were transplanted, demographics, graft function outcomes, ICU stay, DGF rates, 1-y graft, and patient survival and overall patient survival were monitored. For those who remained on dialysis or remained waitlisted on dialysis, patient survival was monitored and compared with the transplanted group using Kaplan-Meir survival curves (MedCalc, Version 22.006).

## RESULTS

### Demographics

Demographic details are provided in Tables 1 and 2. A total of 32 patients were identified who met the criteria for nonvalvular moderate to severe PH (PASP > 50 mm Hg) on pretransplant screening echocardiogram. The mean age of the cohort was 55 ± 10 y. Majority of the patients were AA (22/32; 69%) and males (21/32; 66%). Thirty-one patients (of 32; 97%) were on hemodialysis using an arteriovenous fistula or graft (29/31; 94%), whereas only 1 patient (6%) was maintained on peritoneal dialysis.

**TABLE 1.** Demographics

N	32
Age (mean ± SD), y	55 ± 10
Male sex	21 (66%)
African American Race	22 (69%)
Dialysis modality and access	
Hemodialysis	31 (97%)
Peritoneal dialysis	1 (3%)
Arteriovenous fistula or graft	29 (94%)
Tunneled catheter	2 (6%)

**TABLE 2.** Demographics Comparison

	Transplanted	Not transplanted	*P*
N	17	15	
Age (mean ± SD), y	54 ± 12	56 ± 8	0.58
Male gender	12 (71%)	9 (60%)	0.5
African American Race	10 (59%)	12 (80%)	0.2
Dialysis modality and access			
Hemodialysis	16 (94%)	15 (100%)	
Peritoneal dialysis	1 (6%)	0	
Arteriovenous fistula or graft	14 (82%)	15 (100%)	
PH therapy			
UF only	14 (82%)	15 (100%)	
UF + drugs	1 (6%)		
Drugs alone	2 (12%)		
Listed for transplant	17 (100%)	4 (27%)	
Not listed		11 (73%)	
Non-compliance		5 (45%)	
PH unresponsive to UF and therapy		2 (18%)	
Other		4 (36%)	

PH, pulmonary hypertension; UF, ultrafiltration.

Seventeen (of 32; 53%) patients were transplanted after improvement in PH with the majority undergoing deceased donor kidney transplantation (15/17; 88%), whereas 15 (of 32; 47%) patients remained on dialysis.

Of the 17 patients who were transplanted, 14 (of 17; 82%) responded to UF alone with improvement in PH. One patient (6%) needed additional targeted therapy with phosphodiesterase 5 inhibitors after UF. Two patients (12%) did not have volume components on initial RHC and improved with phosphodiesterase 5 inhibitors alone and were transplanted.

Of the 15 patients (of 32, 47%) who were not transplanted only 4 (27%) remained successfully listed in follow-up, whereas 11 patients were delisted. Five (45%) of patients who were delisted were unable to maintain the dry weight and continued to have heavy intradialytic fluid gains. There was no difference in demographic comparison of those who were transplanted compared with those who remained on dialysis.

### Hemodynamic Outcomes

**Figure 2.**
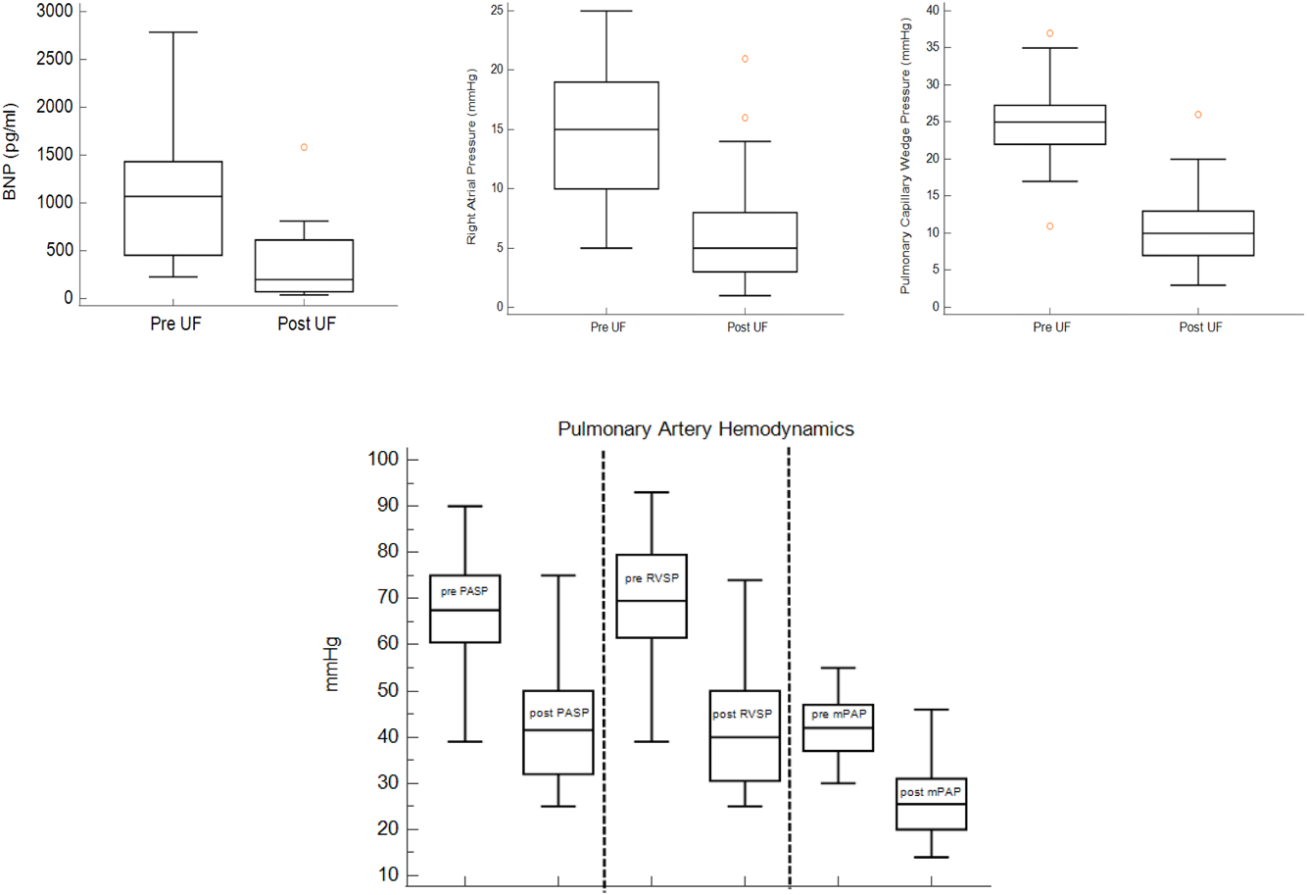
Hemodynamic parameters preultrafiltration and postultrafiltration. BNP, B-type natriuretic peptide; mPAP, mean Pulmonary Artery Pressure; PASP, pulmonary artery systolic pressure; RVSP, right ventricular systolic pressure; UF, ultrafiltration.

These details are provided in Table 3 and Figure 2. Of the 30 patients that required UF, a median of 4 (range, 3–8) UF sessions were required. All measures of volume overload showed a statistically significant decline by univariate analysis. Mean RA pressure declined from 14 ± 5 to 6 ± 5 mm Hg and mean PCWP declined from 24 ± 6 to 10 ± 5 (*P* ≤ 0.0001). Weight declined from 81 ± 17 kg to 77 ± 17 kg (*P* < 0.0001). Seven patients underwent measurement of pre-UF and post-UF measurement of brain natriuretic peptide with again a significant decline from a mean of 1147 ± 861 pg/mL to 370 ± 552 pg/mL (*P* = 0.0045).

**TABLE 3.** Hemodynamics

	Preultrafiltration (mean ± SD)	Postultrafiltration (mean ± SD)	*P* ^*a*^
RA pressure (mm Hg)	14 ± 5	6 ± 5	< 0.0001
PCWP (mm Hg)	24 ± 6	10 ± 5	< 0.0001
Weight (kg)	81 ± 17	77 ± 17	< 0.0001
BNP (pg/mL)	1147 ± 861	370 ± 552	0.0045
PASP (mm Hg)	67 ± 12	43 ± 13	< 0.0001
RVSP (mm Hg)	69 ± 12	43 ± 14	< 0.0001
Mean PAP (mm Hg)	42 ± 7	26 ± 8	< 0.0001
PVR (WU)	3.12 ± 1.6	3.81 ± 2.7	0.28
Blood pressure medications	2.4 ± 1.4	2.15 ± 1.3	0.17

^*a*^Paired T-tests were used for these analyses.

BNP, B-type natriuretic peptide (available only for 7 patients); PAP, pulmonary artery pressure; PASP, pulmonary artery systolic pressure; PCWP, pulmonary capillary wedge pressure; PVR (WU), peripheral vascular resistance (Woods Units); RA, right atrium; RVSP, right ventricular systolic pressure.

Along similar lines, measures of PH also showed a significant improvement by univariate analysis. Pre-UF and post-UF PASP significantly improved from 67 ± 12 to 43 ± 13 mm Hg, RVSP improved from 69 ± 12 to 43 ± 14 mm Hg, and mean PAP improved from 42 ± 7 to 26 ± 8 mm Hg (*P* < 0.0001). Pre-UF and post-UF measures of pulmonary vascular resistance remained unchanged (3.12 ± 1.6 versus 3.81 ± 2.7 WU, *P* = 0.28).

The number of anti-hypertensive medications remained the same before and after UF (2.4 ± 1.4 versus 2.15 ± 1.3, *P* = 0.17).

### Perioperative Outcomes

Seventeen patients underwent successful kidney transplantation. Only 2 patients (12%) of those who were transplanted had a preplanned ICU stay posttransplant considering their surgical risk. The remaining 15 patients (88%) were successfully extubated in the postanesthesia care unit and transferred to step-down level of care. The average length of stay was 5.69 ± 2.87 d posttransplant. The rate of delayed graft function was 59% (10/17) and the 30-day readmission rate was 41% (7/17).

### Graft Outcomes

Seventeen (of 32; 53%) patients were transplanted with majority undergoing deceased donor kidney transplantation (15/17; 88%) at a median time of 140 d (range, 2020–2103) after UF. One year graft survival was 100%. The mean estimated Glomerular Filtration Rate at 3, 6, 9, and 12 mo was 71.56 ± 27 (n = 16), 72.25 ± 28 (n = 13), 74.81 ± 28.83 (n = 16), and 74.9 ± 29.41 (n = 16) mL/min/1.73 m^2^. There were no cases of acute rejection within the first year after transplant.

### Patient Survival

**Figure 3.**
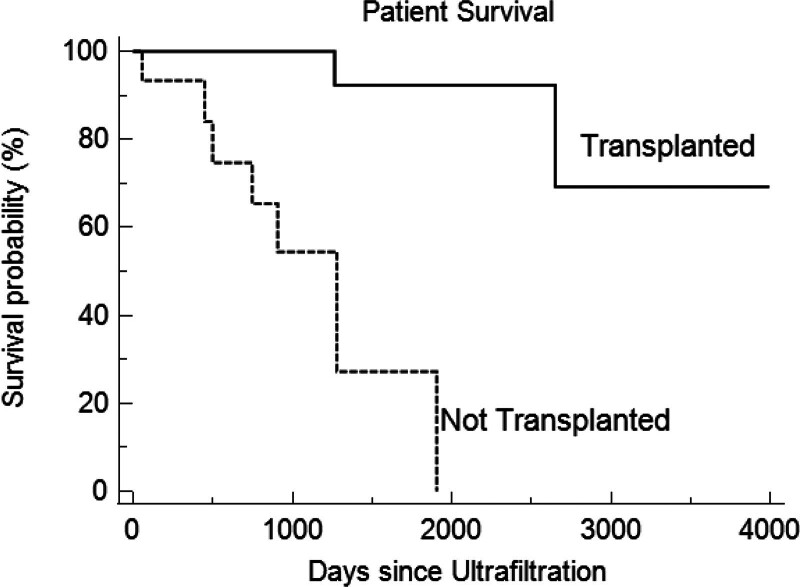
Kaplan-Meier survival curves: transplanted vs not transplanted.

Overall, at a median follow-up of 88 ± 12 mo after UF those who underwent transplant had a significantly higher patient survival of 88% (15/17) as compared with those who remained on dialysis who had a patient survival of 53% (8/15) (*P* = 0.0003; Figure 3).

Of the patients in the transplanted group, 1 died at 6.7 y after transplant from complications of cirrhosis and the other died 2 y posttransplant due to respiratory failure from COVID-19 pneumonia.

## DISCUSSION

The association of pretransplant PH with poor patient survival and graft function has been highlighted in multiple retrospective studies. These studies uniformly find that pretransplant PH is an independent predictor of poor outcomes.^2,7-9^ More recently, a pooled meta-analysis by Brinza et al^10^ included 12 studies and reported a 2-fold higher risk of mortality, DGF, graft failure, graft dysfunction, and graft loss associated with pretransplant PH.

Even though such studies highlight the higher risk associated with transplanting patients with pretransplant PH. It is yet unknown if these risks can be managed or mitigated with pretransplant optimization of PH. Herein, we propose a strategy to manage and mitigate these risks before transplant.

As mentioned earlier the incidence of PH and mortality increases with dialysis vintage.^2^ Therefore, rather than declining these kidney transplant candidates, the next natural step lies in finding an expeditious path to transplant for this group of high-risk patients. Central to our proposed approach is elucidating and correcting volume overload as the cause of PH. Unfortunately, for reasons mentioned earlier volume maybe the major and only modifiable variable. We also feel that approaching and manipulating the AVF/AVG as a source of PH is largely impractical and carries a risk of loss or malfunction of access with no guarantee of kidney transplant soon. The importance of differentiating volume versus intrinsic pulmonary vasculature PH is highlighted in a recent study by Caughey et al^11^ Five-year survival after kidney transplant in patients with pretransplant PH was associated with twice the 5-y mortality (hazard ratio [HR] = 2.11; 95% CI, 1.48-3.03) as compared with those without PH. However, in this study those with evidence of volume overload as the cause of PH had lower mortality after kidney transplant (HR = 1.11; 95% CI, 0.57-2.17) than those without volume overload (HR = 2.87; 95% CI, 1.83-4.49).^11^

In our study, we show that 29 patients of 32 (91%) had improvement in PH with UF alone while only 3 (9%) required PH-directed drug therapy. Therefore, excluding patients from transplant at first glance with a diagnosis of severe PH may prevent them from access to transplant and leave them on dialysis with the higher associated mortality.

In our study, we also add a comparator dialysis group whose survival on dialysis was much worse than after a kidney transplant. It should be noted that many of these patients had subsequent worsening in their dry weight and volume status, after initial optimization. Thus, our findings highlight the urgency with which these recipients need a kidney transplant and stress the importance of long-term partnership with dialysis units in sustaining this optimization till the point of transplant.

Our approach mirrors recent recommendations by Lentine et al^5^ where it is recommended to go a step further in this patient population and attempt to objectively measure, correct, and optimize volume overload as a contributing factor before the kidney transplant.

The major limitation of our study lies in the retrospective single-center design. Reflective of our center patient population, most of the patients were African American thereby limiting the generalizability. The strengths of our study lie in the use of RHC to measure right heart pressures and clearly ascertain precapillary and postcapillary PH. We included only patients with severe PH who are generally excluded from transplant listing or evaluation. Unlike other studies, we have a comparator group of patients who were not transplanted, and their survival outcomes were compared with those who were transplanted.

In this single-center study, we describe an approach to kidney transplantation for patients with severe PH. Significant survival advantages can accrue to candidates who can successfully receive transplants if the perioperative risk can be managed with pretransplant optimization. Patient and dialysis center partnerships are needed to sustain the initial benefits of aggressive UF, so that transplant candidacy can be maintained for this high-risk population.
